# Star product on complex sphere $\mathbb {S}^{2n}$

**DOI:** 10.1007/s11005-018-1074-z

**Published:** 2018-03-22

**Authors:** A. Mudrov

**Affiliations:** Department of Mathematics, University of Leicester, University Road, Leicester, LE1 7RH UK

**Keywords:** Quantum groups, Star product, Even spheres, Verma modules, 17B10, 17B37, 53D55

## Abstract

We construct a $U_q\bigl (\mathfrak {s}\mathfrak {o}(2n+1)\bigr )$-equivariant local star product on the complex sphere $\mathbb {S}^{2n}$ as a non-Levi conjugacy class $SO(2n+1)/SO(2n)$.

## Introduction

In this paper, we incorporate an example of homogeneous space with non-Levi stabilizer into a uniform quantization scheme for closed conjugacy classes of simple algebraic groups. Originally, this approach was developed in 2003 for Levi classes and utilized the presence of quantum isotropy subgroup in the total quantum group, [1, 2]. The key distinction of non-Levi classes is the absence of a natural candidate for such a subgroup because its root basis cannot be made a part of the total root basis. Still the coordinate ring of the class can be quantized by an operator realization on certain modules [3]. Such a quantization is formulated in terms of generators and relations and is not apparently local. On the other hand, a dynamical twist constructed from the Shapovalov form yields a local version of the star product on Levi classes [1, 2] (see also [4, 5] for coadjoint orbits with the Kirillov bracket). It is natural to extend that approach to all closed conjugacy classes. Such a possibility for $\mathbb {S}^4$ was pointed out without proof in [6]. Here we give a solution for all even-dimensional spheres.

Sphere is a relatively simple curved space endowed with a rich structure that has numerous applications. An interest to its quantum version started to grow with the invention of quantum groups [7], and $\mathbb {S}^{2}_q$ was the first quantum *G*-space [8] after Manin’s $\mathbb {C}^2_q$ [9]. A review of various constructions of the q-sphere in small dimensions and some references to its applications can be found in [10].

An even sphere admits several independent although isomorphic equivariant quantizations: a subvariety of the quantum Euclidean plane [11], an induced representation of a quantum symmetric pair (cf. [12]), and a subalgebra of linear operators on a highest weight module of the orthogonal quantum group [13]. Each particular reincarnation has its pluses that help tackling hard issues arising in other approaches. For instance, the operator realization of $\mathbb {C}_q[\mathbb {S}^{2n}]$ allows to study representations of the coideal subalgebra in the corresponding symmetric pair [12]. All realizations of $\mathbb {C}_q[\mathbb {S}^{2n}]$ known to date appeal to generators and relations. At the same time, a local formulation may be of interest for some applications, like Fedosov’s star product approach to the index theorem [14]. The present work fills that gap. Note that, like in the Levi case [1], this problem can be placed in a more general context of quantum vector bundles addressed in [12]. This is also a part of the Gelfand–Zetlin reduction for orthogonal quantum groups, that is open by now. It turns out that local quantization of the function algebra on $\mathbb {S}^{2n}$ (the trivial bundle) can be done with elementary means and deserves a special consideration.

The original approach to the star product on Levi classes was as follows. Let $\mathfrak {k}\subset \mathfrak {g}$ be the isotropy Levi subalgebra of a point *t* and $\mathfrak {p}_\pm \subset \mathfrak {g}$ its parabolic extensions. One associates with *t* a certain weight $\lambda \in \mathfrak {h}^*$ and a pair of modules $M_\lambda , N_\lambda$ of, respectively, highest and lowest weights $\lambda$ and $-\,\lambda$. There is a (essentially unique) $U_q(\mathfrak {g})$-invariant form $M_\lambda \otimes N_\lambda \rightarrow \mathbb {C}$, which is non-degenerate if and only if the modules are irreducible. In that case, there exists the inverse form $\mathbb {C}\rightarrow N_\lambda \otimes M_\lambda$ and its lift assigning $1\mapsto \mathcal {F}\in U_q(\mathfrak {p}_+)\otimes U_q(\mathfrak {p}_-)$ (a completed tensor product). The element $\mathcal {F}$ gives rise to a “bidifferential” operator via the left coregular action on the Hopf dual $\mathcal {A}=U_q^*(\mathfrak {g})$. With this operator, the multiplication in $\mathcal {A}$ is twisted to a non-associative operation invariant under the right coregular action of $U_q(\mathfrak {g})$. The key observation is that the new multiplication becomes associative when restricted to the subspace $\mathcal {A}^\mathfrak {k}$ of $U_q(\mathfrak {k})$-invariants in $\mathcal {A}$. As a (right) $U_q(\mathfrak {g})$-module, $\mathcal {A}^\mathfrak {k}$ has the same structure as the $U(\mathfrak {g})$-module $\mathbb {C}[G/K]$, where $K\subset G$ is the centralizer subgroup of the point *t*. Hence, $\mathcal {A}^\mathfrak {k}$ is a flat deformation of $\mathbb {C}[G/K]$. It is known that the initial star product on $\mathcal {A}$ is local [15]; therefore, the resulting multiplication is local as well.

In the non-Levi case, one can go along those lines and define $\mathcal {A}^\mathfrak {k}$ as the joint kernel of certain operators that deform generators of $\mathfrak {k}$. Then the new product will be associative on $\mathcal {A}^\mathfrak {k}$ as in the Levi case [16]. However, those operators do not close up to a deformation of $U_q(\mathfrak {k})$, so one cannot be sure that $\mathcal {A}^\mathfrak {k}$ has the proper size. (Observe that kernel can decrease under deformation.) Therefore, the problem is to check the size of $\mathcal {A}^\mathfrak {k}$. We do it for $\mathbb {S}^{2n}$ via a harmonic analysis relative to quantized $SO(2n+1)$. Note that odd-dimensional spheres belong to the second connected component of the orthogonal group *O*(2*n*), and the current methods are not directly applicable.

The paper consists of five sections: after Introduction, we recall quantization of $\mathbb {C}[\mathbb {S}^{2n}]$ via operator realization on a highest weight module $M_\lambda$ in Sect. 2. In Sect. 3 we construct a system of vectors that spans $M_\lambda$. We prove it to be a basis in Sect. 4 by computing the Shapovalov form on $M_\lambda$. This way, we show that $M_\lambda$ is irreducible and the form is invertible. In the final section we show that for finite $U_q(\mathfrak {g})$-module $V_q$, the dimension of $V^\mathfrak {k}_q$ is equal to $\dim V^\mathfrak {k}$ of the classical $\mathfrak {k}$-invariants. We do it via realization of $V_q$ with $\dim V_q^\mathfrak {k}>0$ in the coordinate ring of the quantum Euclidean plane $\mathbb {C}^{2n+1}_q$.

## Operator realization of $\mathbb {C}_q[\mathbb {S}^{2n}]$

Throughout the paper, $\mathfrak {g}$ stands for the Lie algebra $\mathfrak {s}\mathfrak {p}(2n+1)$. We are looking for quantization of the polynomial ring $\mathbb {C}[\mathbb {S}^{2n}]$ that is invariant under an action of the quantized universal enveloping algebra $U_q(\mathfrak {g})$. We regard $\mathbb {S}^{2n}$ as a conjugacy class of the Poisson group $G=SO(2n+1)$ equipped with the Drinfeld–Sklyanin bracket corresponding to the standard solution $r\in \mathfrak {g}\otimes \mathfrak {g}$ of the classical Yang–Baxter equation [7]. The group *G* supports the Semenov–Tian–Shansky bivector field

2.1 $$\begin{aligned} r_-^{l,l}+r_-^{r,r}-r_-^{r,l}-r_-^{l,r}+r_+^{r,l}-r_+^{l,r}, \end{aligned}$$

making it a Poisson *G*-space with respect to conjugation. Here $r_-$ and $r_+$ are, respectively, the skew-symmetric and invariant symmetric parts of *r*, and the superscripts designate the vector fields

$$\begin{aligned} \left( \xi ^l f\right) (g)=\frac{\mathrm{d}}{\mathrm{d}t}f\left( ge^{t\xi }\right) |_{t=0}, \quad (\xi ^r f)(g)=\frac{\mathrm{d}}{\mathrm{d}t}f\left( e^{t\xi }g\right) |_{t=0}, \end{aligned}$$

where $\xi \in \mathfrak {g}$ and *f* is a smooth function on *G*. This bivector field (2.1) is tangent to every conjugacy class of *G*. In particular, the sphere $\mathbb {S}^{2n}$ becomes a homogeneous Poisson manifold over the Poisson group *G* [17].

Quantization of $\mathbb {C}[G]$ along (2.1) gives rise to the reflection equation dual of $U_q(\mathfrak {g})$ [18]. Accordingly, the algebra $\mathbb {C}_q[\mathbb {S}^{2n}]$ can be presented as its quotient. Here we recall that construction.

Let $\mathfrak {h}\subset \mathfrak {g}$ denote the Cartan subalgebra equipped with the inner product restricted from an $\mathrm {ad}$-invariant form on $\mathfrak {g}$. We endow the dual space $\mathfrak {h}^*$ with the inverse form (., .) and normalize it so that short roots have length 1. For any $\mu \in \mathfrak {h}^*$ we denote by $h_\mu \in \mathfrak {h}$ the vector such that $\nu (h_\mu )=(\nu ,\mu )$ for all $\nu \in \mathfrak {h}^*$.

The root system $\mathrm {R}$ contains an orthonormal basis $\Lambda =\{\varepsilon _i\}_{i=1}^n\subset \mathfrak {h}^*$ of short roots. We choose the basis of simple positive roots $\Pi$ as $\alpha _1=\varepsilon _1, \alpha _{i}=\varepsilon _{i}-\varepsilon _{i-1}, i=2,\ldots , n$. We define the subalgebra $\mathfrak {l}\simeq \mathfrak {g}\mathfrak {l}(n)\subset \mathfrak {g}$ of maximal rank with the root basis $\Pi _\mathfrak {l}=\{\alpha _i\}_{i=2}^n$.

Throughout the paper we assume that $q\in \mathbb {C}$ is not a root of unity and use the notation $\bar{q}=q^{-1}$, $[z]_q=\frac{q^z-q^{-z}}{q-q^{-1}}$, and $[x,y]_a=xy-ayx$ for $a\in \mathbb {C}$. The quantum group $U_q(\mathfrak {g})$ is a $\mathbb {C}$-algebra generated by $q^{\pm h_\alpha }, e_{\pm \alpha }, \alpha \in \Pi$, such that $q^{h_{\alpha }}e_{\pm \beta }q^{- h_{\alpha }}= q^{\pm (\alpha ,\beta )} e_{\pm \beta }$ and $[e_{\alpha },e_{-\beta }]=\delta _{\alpha ,\beta }[h_\alpha ]_q$ for all $\alpha ,\beta \in \Pi$. The generators $e_{\pm \alpha }$ satisfy the q-Serre relations

$$\begin{aligned} \left[ e_{\pm \alpha },[e_{\pm \alpha },e_{\pm \beta }]_q\right] _{\bar{q}}=0, \quad \forall \alpha ,\beta \in \Pi \quad \text{ s.t. }\quad \frac{2(\alpha ,\beta )}{(\alpha ,\alpha )}=-\,1, \quad \text{ and } \quad [e_{\pm \alpha _1},e_{\pm \delta }]=0, \end{aligned}$$

where $e_{\pm \delta }=[e_{\pm \alpha _1},[e_{\pm \alpha _1},e_{\pm \alpha _2}]_q]_{\bar{q}}$. Also, $[e_{\pm \alpha },e_{\pm \beta }]=0$ once $(\alpha ,\beta )=0$ [7].

The subset $\Pi _\mathfrak {k}=\{\delta ,\alpha _1,\ldots ,\alpha _n\}\subset \mathrm {R}^+$ forms a root basis for a subalgebra $\mathfrak {k}\subset \mathfrak {g}$ isomorphic to $\mathfrak {s}\mathfrak {o}(2n)$. Although $e_{\pm \delta }$ are deformations of classical root vectors, they do not generate an $\mathfrak {s}\mathfrak {l}(2)$-subalgebra in $U_q(\mathfrak {g})$, so we have no natural subalgebra $U_q(\mathfrak {k})$ in $U_q(\mathfrak {g})$. Still $e_{\pm \delta }$ play a role in what follows.

By $U_q(\mathfrak {h})\subset U_q(\mathfrak {g})$ we denote the subalgebra generated by $\{q^{\pm h_\alpha }\}_{\alpha \in \Pi }$. We use the notation $\mathfrak {g}_\pm \subset \mathfrak {g}$ for the Lie subalgebras generated by $\{e_{\pm \alpha }\}_{\alpha \in \Pi }$. They generate subalgebras $U_q(\mathfrak {g}_\pm )\subset U_q(\mathfrak {g})$.

Fix the weight $\lambda \in \mathfrak {h}^*$ by the conditions $q^{2(\lambda ,\varepsilon _i)}=-\,q^{-1}$ for all $i=1,\ldots ,n$, and $(\alpha _i, \lambda )=0$ for $i>1$. Define two one-dimensional representations $\mathbb {C}_{\pm \lambda }$ of $U_q(\mathfrak {l})$ by $e^{h_\alpha }\mapsto q^{\pm (\lambda ,\alpha )}, \alpha \in \Pi _\mathfrak {g}$, and by zero on the generators on nonzero weight. Extend them to representations of $U_q(\mathfrak {p}_\pm )$ by zero on $e_{\pm \alpha }$ for all $\alpha \in \Pi _\mathfrak {g}$. Then set

$$\begin{aligned} \hat{M}_\lambda =U_q(\mathfrak {g})\otimes _{U_q(\mathfrak {p}_+)} \mathbb {C}_\lambda , \quad \hat{N}_\lambda =U_q(\mathfrak {g})\otimes _{U_q(\mathfrak {p}_-)} \mathbb {C}_{-\lambda }. \end{aligned}$$

Denote by $1_\lambda \in M_\lambda$ and $1^*_\lambda \in N_\lambda$ their highest/lowest weight generators. Due to the special choice of $\lambda$, the vectors $e_{-\delta }1_\lambda \in \hat{M}_\lambda$ and $e_{\delta }1^*_\lambda \in \hat{N}_\lambda$ are killed by $e_\alpha$ and, respectively, by $e_{-\alpha }$ for all $\alpha \in \Pi$. They generate submodules $\hat{M}_{\lambda -\delta }\subset M_\lambda$ and $\hat{N}_{\lambda -\delta }\subset N_\lambda$. Set $M_{\lambda }=\hat{M}_\lambda /\hat{M}_{\lambda -\delta }$ and $N_\lambda =\hat{N}_{\lambda }/\hat{N}_{\lambda -\delta }$.

The module $M_\lambda$ supports quantization of $\mathbb {C}[\mathbb {S}^{2n}]$ in the following sense. The sphere $\mathbb {S}^{2n}$ is isomorphic to a subvariety in *G* of orthogonal matrices with eigenvalues $\pm 1$, where 1 is multiplicity-free. It is a conjugacy class with a unique point of intersection with the maximal torus relative to $\mathfrak {h}$. The isotropy subalgebra of this point is $\mathfrak {k}$. Quantization of $\mathbb {C}[G]$ along the Poisson bracket (2.1) can be realized as a subalgebra $\mathbb {C}_q[G]\subset U_q(\mathfrak {g})$ invariant under the adjoint action. The image of $\mathbb {C}_q[G]$ in $\mathrm {End}(M_\lambda )$ is an equivariant quantization of $\mathbb {C}[\mathbb {S}^{2n}]$, see [13] for details.

## Spanning $M_\lambda$

In this section we introduce a set of vectors in $M_\lambda$ which is proved to be a basis in the subsequent section. Here we demonstrate that it spans $M_\lambda$. Put $f_{\alpha }=e_{-\alpha }$ for all simple roots and define

$$\begin{aligned} f_{\varepsilon _i}=\left[ \ldots [f_{\alpha _1},f_{\alpha _2}]_{\bar{q}},\ldots f_{\alpha _i}\right] _{\bar{q}}\in U_q(\mathfrak {g}_-), \quad 1\leqslant i\leqslant n. \end{aligned}$$

The elements $f_{\varepsilon _i}$ can be included in the set of composite root vectors generating a Poincare–Birkhoff–Witt basis in $U_q(\mathfrak {g}_-)$, [19]. By deformation arguments, the set of monomials $\mathcal {B}=\{f_{\varepsilon _1}^{m_{_1}}\ldots f_{\varepsilon _n}^{m_{_n}}1_\lambda \}_{m_1,\ldots ,m_n\in \mathbb {Z}_+}$ is a basis in $M_\lambda$ extended over the local ring $\mathbb {C}[[\hbar ]]$, where $\hbar =\log q$, see [13] and references therein. We will prove that $\mathcal {B}$ is a $\mathbb {C}$-basis once *q* is not a root of unity.

Let $\mathfrak {k}_m^-$ denote the subspace $\mathbb {C}f_\delta +\mathrm {Span}\{f_{\alpha _2},\ldots ,f_{\alpha _m}\}\subset U_q(\mathfrak {g}_-)$ assuming $m\geqslant 2$.

### Lemma 3.1

For all $1<i\leqslant m$, the elements $f_{\varepsilon _i}$ belong to the normalizer of the left ideal $U_q(\mathfrak {g})\mathfrak {k}_m^-$.

### Proof

The Serre relations readily yield $[f_{\alpha _m}, f_{\varepsilon _m}]_{\bar{q}}=0$, for $m>1$. For $1<i<m$, the identity $[f_{\alpha _i}, f_{\varepsilon _m}]=0$ follows from Lemma A.1. So we are left to study how $f_{\varepsilon _i}$ commute with $f_\delta$.

It is immediate that $[f_\delta ,f_{\varepsilon _2}]=0$, cf. [6], which completes the proof for $m=2$. Suppose that $m=3$. All calculations below are done modulo $U_q(\mathfrak {g})\mathfrak {k}_m^-$. Denote $a=[2]_q$, then

3.2 $$\begin{aligned} f_{\alpha _1}f_{\alpha _2}f_{\alpha _1}= & {} \frac{1}{a}f_{\alpha _2}f_{\alpha _1}^{2} ,\nonumber \\ f_{\alpha _2}f_{\alpha _1}^3= & {} \left( a-\frac{1}{a}\right) f_{\alpha _1}f_{\alpha _2}f_{\alpha _1}^2 ,\quad f_{\alpha _2}f_{\alpha _1}^3=\left( a^2-1\right) f_{\alpha _1}^2f_{\alpha _2}f_{\alpha _1}, \end{aligned}$$

where the left equality means $f_\delta \in U_q(\mathfrak {g})\mathfrak {k}_3^-$ and the last two equalities are obtained from it and from $[f_\delta ,f_{\alpha _1}]=0$ (a Serre relation). Furthermore, the Serre relations along with (3.2) yield

$$\begin{aligned} f_{\alpha _1}f_{\alpha _2}(f_{\alpha _1} f_{\alpha _3})f_{\alpha _2}f_{\alpha _1}= & {} f_{\alpha _1}f_{\alpha _2} f_{\alpha _3}(f_{\alpha _1} f_{\alpha _2}f_{\alpha _1})= \frac{1}{a} f_{\alpha _1}(f_{\alpha _2} f_{\alpha _3}f_{\alpha _2})f_{\alpha _1}^2\\= & {} \frac{1}{a^2} f_{\alpha _3}(f_{\alpha _1}f_{\alpha _2}^2)f_{\alpha _1}^2 \\= & {} \frac{1}{a}f_{\alpha _3}f_{\alpha _2}(f_{\alpha _1}f_{\alpha _2}f_{\alpha _1}^2)\\&-\frac{1}{a^2}f_{\alpha _3}f_{\alpha _2}^2f_{\alpha _1}^3=\frac{1}{a^2-1}f_{\alpha _3} f_{\alpha _2}^2f_{\alpha _1}^3-\frac{1}{a^2} f_{\alpha _3}f_{\alpha _2}^2f_{\alpha _1}^3 \\= & {} \frac{1}{a^2(a^2-1)}f_{\alpha _3}f_{\alpha _2}^2f_{\alpha _1}^3,\\ f_{\alpha _2}(f_{\alpha _1}^2 f_{\alpha _3})f_{\alpha _2}f_{\alpha _1}= & {} f_{\alpha _2} f_{\alpha _3} (f_{\alpha _1}^2 f_{\alpha _2}f_{\alpha _1}) =\frac{1}{(a^2-1)}f_{\alpha _2} f_{\alpha _3} f_{\alpha _2}f_{\alpha _1}^3. \end{aligned}$$

Multiply the first equality by *a* and subtract from the second:

$$\begin{aligned} f_\delta f_{\varepsilon _3}= & {} \left( f_{\alpha _2}f_{\alpha _1}^2 - af_{\alpha _1}f_{\alpha _2}f_{\alpha _1}\right) f_{\alpha _3}f_{\alpha _2}f_{\alpha _1}= \frac{1}{a(a^2-1)}\left( af_{\alpha _2} f_{\alpha _3} f_{\alpha _2}-f_{\alpha _3}f_{\alpha _2}^2\right) f_{\alpha _1}^3\nonumber \\\in & {} U_q(\mathfrak {g})\mathfrak {k}_3^-. \end{aligned}$$

This completes the case $m=3$.

For all $i>k\geqslant 1$, the elements $f_{\varepsilon _i-\varepsilon _k}=[f_{\alpha _{k+1}},\ldots [f_{\alpha _{i-1}},f_{\alpha _i}]_{\bar{q}}\ldots ]_{\bar{q}}$ belong to $\in U_q(\mathfrak {g})\mathfrak {k}_m^-$ with $m\geqslant i$. Then $f_\delta f_{\varepsilon _i}=-q^{-1}f_\delta f_{\varepsilon _i-\varepsilon _3}f_{\varepsilon _3}=-q^{-1}f_{\varepsilon _i-\varepsilon _3}f_\delta f_{\varepsilon _3}=0 \> \mathrm {mod}\> U_q(\mathfrak {g})\mathfrak {k}_m^-,$ for all $m\geqslant i>3$, as required. $\square$

### Corollary 3.2

The set $\mathcal {B}$ spans $M_\lambda$. The action of $U_q(\mathfrak {g}_-)$ on $M_\lambda$ is given by

$$\begin{aligned} f_{\alpha _1} f_{\varepsilon _1}^{m_1}\ldots f_{\varepsilon _n}^{m_n}1_\lambda= & {} f_{\varepsilon _1}^{m_1+1} f_{\varepsilon _{2}}^{m_{2}} \ldots f_{\varepsilon _n}^{m_n}1_\lambda ,\\ f_{\alpha _{i+1}} f_{\varepsilon _1}^{m_1}\ldots f_{\varepsilon _n}^{m_n}1_\lambda= & {} -\,q[m_{i}]_qf_{\varepsilon _1}^{m_1}\ldots f_{\varepsilon _{i}}^{m_{i}-1} f_{\varepsilon _{i+1}}^{m_{i+1}+1} \ldots f_{\varepsilon _n}^{m_n}1_\lambda , \quad i>1. \end{aligned}$$

### Proof

First let us show that $f_{\varepsilon _{i+1}}f_{\varepsilon _{i}}= q^{-1}f_{\varepsilon _{i}}f_{\varepsilon _{i+1}}\mod U_q(\mathfrak {g})\mathfrak {k}^-_{i+1}$. Indeed, for $i=1$ we have $f_{\varepsilon _2}f_{\varepsilon _1}= q^{-1} f_{\varepsilon _1}f_{\varepsilon _2}- q^{-1} f_\delta =q^{-1} f_{\varepsilon _1}f_{\varepsilon _2}\mod U_q(\mathfrak {g})\mathfrak {k}_2^-$ as required. For $i>2$ we get

$$\begin{aligned} \left[ f_{\varepsilon _i},f_{\varepsilon _{i+1}}\right] _q=\left[ \left[ f_{\varepsilon _1},f_{\varepsilon _i-\varepsilon _2}\right] _{\bar{q}},f_{\varepsilon _{i+1}}\right] _q =\left[ f_{\varepsilon _1},\left[ f_{\varepsilon _i-\varepsilon _2},f_{\varepsilon _{i+1}}\right] \right] +\left[ \left[ f_{\varepsilon _1}, f_{\varepsilon _{i+1}}\right] _q,f_{\varepsilon _i-\varepsilon _2}\right] _{\bar{q}}. \end{aligned}$$

The first summand vanishes since $f_{\varepsilon _i-\varepsilon _2}$ commutes with $f_{\varepsilon _{i+1}}$, by Lemma A.1. The internal commutator in the second summand is $[f_\delta ,f_{\varepsilon _{i+1}-\varepsilon _2}]_{\bar{q}}\in U_q(\mathfrak {g})\mathfrak {k}^-_{i+1}$, so this term is in $U_q(\mathfrak {g})\mathfrak {k}^-_{i+1}$ as well.

Now we can complete the proof. The linear span $\mathbb {C}\mathcal {B}$ is invariant under the obvious action of $f_{\alpha _1}=f_{\varepsilon _1}$. For $i>0$, we push $f_{\alpha _{i+1}}$ to the right in the product

$$\begin{aligned} f_{\alpha _{i+1}}f_{\varepsilon _1}^{m_1}\ldots f_{\varepsilon _n}^{m_n}1_\lambda =f_{\varepsilon _1}^{m_1}\ldots f_{\alpha _{i+1}}f_{\varepsilon _{i}}^{m_i}\ldots f_{\varepsilon _n}^{m_n}1_\lambda . \end{aligned}$$

Thanks to Lemma 3.1, we replace $f_{\alpha _{i+1}}f_{\varepsilon _{i}}^{m_i}$ with

$$\begin{aligned} \left[ f_{\alpha _{i+1}},f_{\varepsilon _{i}}^{m_i}\right] _{q^{m_i}}= & {} -\,q\sum _{l=0}^{m_i-1}q^lf_{\varepsilon _{i}}^lf_{\varepsilon _{i+1}}f_{\varepsilon _{i}}^{m_i-1-l}\nonumber \\= & {} -\,q[m_i]_q f_{\varepsilon _{i}}^{m_i-1}f_{\varepsilon _{i+1}} \mod U_q(\mathfrak {g})\mathfrak {k}^-_{j+1}. \end{aligned}$$

For any form $\Phi$ in $n-i$ variables, the ideal $U_q(\mathfrak {g})\mathfrak {k}^-_{j+1}\subset U_q(\mathfrak {g})\mathfrak {k}^-_{n}$ kills $\Phi (f_{\varepsilon _{i+1}},\ldots , f_{\varepsilon _n})1_\lambda$ by Lemma  3.1. This yields the action of $f_{\alpha _{i+1}}$ on $\mathbb {C}\mathcal {B}$ and proves its $U_q(\mathfrak {g}_-)$-invariance. Since $\mathbb {C}\mathcal {B}\ni 1_\lambda$, it coincides with $M_\lambda$. $\square$

## Invariant bilinear form $M_\lambda \otimes N_\lambda \rightarrow \mathbb {C}$

Introduce positive root vectors by

$$\begin{aligned} e_{\varepsilon _i}=[\ldots [e_{\alpha _{i}},e_{\alpha _{i-1}}]_q,\ldots e_{\alpha _1}]_{q}, \quad 1\leqslant i\leqslant n. \end{aligned}$$

The elements $f_{\varepsilon _i},e_{\varepsilon _i}$ are known to generate $U_q(\mathfrak {s}\mathfrak {l}(2))$-subalgebras in $U_q(\mathfrak {g})$ with the commutation relation $[e_{\varepsilon _i},f_{\varepsilon _i}]=[h_{\varepsilon _i}]_q$. Define by induction $\tilde{e}_{\varepsilon _{i+1}}=[e_{\alpha _{i+1}},\tilde{e}_{\varepsilon _{i}}]_{\bar{q}}$ with $\tilde{e}_{\varepsilon _1}=e_{\alpha _1}$. Then $\omega (f_{\varepsilon _i})=\tilde{e}_{\varepsilon _i}$, where $\omega$ is the anti-algebra involution of $U_q(\mathfrak {g})$ extending $\omega (f_\alpha )= e_\alpha , \forall \alpha \in \Pi$.

Fix the comultiplication on $U_q(\mathfrak {g})$ as in [19]:

$$\begin{aligned} \Delta (e_{\alpha })=e_{\alpha }\otimes q^{h_\alpha }+1\otimes e_{\alpha }, \quad \Delta (f_{\alpha })=f_{\alpha }\otimes 1+q^{-h_\alpha }\otimes f_{\alpha }, \end{aligned}$$

and $\Delta (q^{h_\alpha })=q^{h_\alpha }\otimes q^{h_\alpha }$, for all $\alpha \in \Pi$. Then $\gamma ^{-1}(e_{\alpha })=-q^{-h_{\alpha }}e_\alpha$ for the inverse antipode $\gamma ^{-1}$. Define a map $U_q(\mathfrak {g})\rightarrow \mathbb {C}, x\mapsto \langle x\rangle$, as the composition of the projection $U_q(\mathfrak {g})\rightarrow U_q(\mathfrak {h})\mod \sum _{\alpha \in \Pi }(e_{-\alpha }U_q(\mathfrak {g})+U_q(\mathfrak {g})e_{\alpha })$ with evaluation at $\lambda$. The assignment $(x 1_\lambda ,y1^* _\lambda )= \langle \gamma ^{-1}(y)x\rangle$, $\forall x,y\in U_q(\mathfrak {g})$, defines a unique invariant bilinear form $M_\lambda \otimes N_\lambda \rightarrow \mathbb {C}$ such that $(1_\lambda ,1^*_\lambda )=1$.

### Lemma 4.1

Suppose that $k_i,m_i \in \mathbb {Z}_+$, for $i=1,\ldots ,n$. Then

4.3 $$\begin{aligned} \langle e_{\varepsilon _n}^{k_n}\ldots e_{\varepsilon _1}^{k_1} f_{\varepsilon _1}^{m_1}\ldots f_{\varepsilon _n}^{m_n}\rangle =\prod _{i=1}^{n}\delta _{k_i,m_i} [m_i]_q!\theta ^{-m_i}q^{-m_i(\lambda ,\varepsilon _i)-\frac{m_i}{2}}(-1)^{m_i}, \end{aligned}$$

where $$\theta =q^{\frac{1}{2}}-q^{-\frac{1}{2}}$$.

### Proof

The $\delta$-symbols are due to orthogonality of weight subspaces $M_\lambda [\mu ]$ and $N_\lambda [\nu ]$ unless $\mu =-\,\nu$. Now we prove factorization of the matrix coefficients on setting $k_i=m_i$ for all *i*. Observe that

4.4 $$\begin{aligned} e_{\varepsilon }^{m} f_{\varepsilon }^{m} = \prod _{l=1}^m\frac{[\frac{l}{2}]_q}{[\frac{1}{2}]_q} \prod _{l=0}^{m-1}\left[ h_{\varepsilon }-\frac{l}{2}\right] _q \mod U_q(\mathfrak {g})e_{\varepsilon }, \quad \forall \varepsilon \in \Lambda , \end{aligned}$$

and $\langle e_{\varepsilon }^{m} f_{\varepsilon }^{m}\rangle =(-\,1)^mq^{-m(\lambda ,\varepsilon )-\frac{m}{2}}\theta ^{-m}[m]_q!$ on substitution $q^{2(\lambda ,\varepsilon )}=-\,q^{-1}$. Suppose we have proved that the LHS of (4.3) is equal to $\langle e_{\varepsilon _n}^{m_n}\ldots e_{\varepsilon _s}^{m_s} f_{\varepsilon _s}^{m_s}\ldots f_{\varepsilon _n}^{m_n}\rangle \prod _{l=1}^{s-1}\langle e_{\varepsilon _{l}}^{m_{l}} f_{\varepsilon _{l}}^{m_{l}}\rangle$ for some $s=1,\ldots , n-1$. For all $i>s, [e_{\varepsilon _s},f_{\varepsilon _i}]\in U_q(\mathfrak {g})\mathfrak {k}^-_{i}\subset U_q(\mathfrak {g})\mathfrak {k}^-_{n}$. Then $e_{\varepsilon _s}\ldots f_{\varepsilon _{s+1}}^{m_{s+1}}\ldots f_{\varepsilon _n}^{m_n}1_\lambda =0$, by Lemma  3.1. Now the presentation (4.4) for $\varepsilon =\varepsilon _s$, along with the orthogonality of different $\varepsilon _i$, gives $\langle e_{\varepsilon _n}^{m_n}\ldots e_{\varepsilon _{s-1}}^{m_{s-1}} f_{\varepsilon _{s-1}}^{m_{s-1}}\ldots f_{\varepsilon _n}^{m_n}\rangle \prod _{l=1}^{s}\langle e_{\varepsilon _{l}}^{m_{l}} f_{\varepsilon _{l}}^{m_{l}}\rangle$. Induction on *s* completes the proof. $\square$

There is also an $\omega$-contravariant form on $M_\lambda$ defined by $x1_\lambda \otimes y1_\lambda \mapsto \langle \omega (x)y\rangle$, for all $x,y\in U_q(\mathfrak {g})$. It is called the Shapovalov form and related to the invariant form in the obvious way.

### Proposition 4.2

Suppose that *q* is not a root of unity. Then

1. $\mathcal {B}\subset M_\lambda$ is an orthogonal (non-normalized) basis with respect to the Shapovalov form.
2. The modules $M_\lambda$ and $N_\lambda$ are irreducible.
3. The tensor
  $$\begin{aligned} \mathcal {F}=\sum _{m_1,\ldots ,m_n=0}^{\infty }(-\theta )^{\sum _{i=1}^{n}m_i}\> \frac{\prod _{i=1}^{n}q^{-\frac{m_i^2}{2}+2m_i(i-1)}}{\prod _{i=1}^n[m_i]_{q}!} \tilde{e}_{\varepsilon _1}^{m_1}\ldots \tilde{e}_{\varepsilon _n}^{m_n} \otimes f_{\varepsilon _1}^{m_1}\ldots f_{\varepsilon _n}^{m_n} \end{aligned}$$
  is a lift of the inverse invariant form, $\mathbb {C}\rightarrow N_\lambda \otimes M_\lambda \rightarrow U_q(\mathfrak {g}_+)\otimes U_q(\mathfrak {g}_-), 1\mapsto \mathcal {F}$.

### Proof

(1) Corollary 3.2 with (4.3) proves the completeness of $\mathcal {B}$ and independence. All weight subspaces in $M_\lambda$ have dimension 1, and the form is non-degenerate; hence, the basis $\mathcal {B}$ is orthogonal with respect to the Shapovalov form. (2) Non-degeneracy of the form implies irreducibility of $M_\lambda$. (3) The normalizing coefficients in $\mathcal {F}$ are obtained from (4.3) via the equality $(x 1_\lambda ,\gamma (y)1^*_\lambda )= \langle y x \rangle$ and $\gamma (e_{\varepsilon _i})=-\,\tilde{e}_{\varepsilon _i}q^{-h_{\varepsilon _i}+2(i-1)}$ for all $i=1,\ldots ,n$. $\square$

## Star product on $\mathbb {S}^{2n}$

Denote by $\mathcal {A}_q$ the RTT dual of $U_q(\mathfrak {g})$ with multiplication $\bullet$ and the Hopf paring (., .). It is equipped with the two-sided action (here $f^{(1)}\otimes f^{(2)}=\Delta (f)$ in the Sweedler notation)

$$\begin{aligned} u \triangleright f=f^{(1)}\left( f^{(2)},u\right) , \quad f\triangleleft u=\left( f^{(1)},u\right) f^{(2)}, \quad \forall u\in U_q(\mathfrak {g}), \quad f \in \mathcal {A}_q. \end{aligned}$$

making it a $U_q(\mathfrak {g})$-bimodule algebra. The multiplication $\bullet$ is known to be local [15]. We define a new operation $\star$ by

5.5 $$\begin{aligned} f\star g= (\mathcal {F}_1\triangleright f )\bullet (\mathcal {F}_2 \triangleright g), \quad f,g \in \mathcal {A}_q. \end{aligned}$$

It is obviously equivariant with respect to the right coregular action of $U_q(\mathfrak {g})$. However, $\star$ is not associative on the entire $\mathcal {A}_q$.

As a two-sided $U_q(\mathfrak {g})$-module, $\mathcal {A}_q$ is isomorphic to $\oplus _V V^*\otimes V$, where the summation is over all equivalence classes of irreducible finite-dimensional representations of $U_q(\mathfrak {g})$. This is a q-version of the Peter–Weyl decomposition.

For every $U_q(\mathfrak {g})$-module *V* we define $V^\mathfrak {k}\subset V$ to be the intersection of the space $V^\mathfrak {l}$ of $U_q(\mathfrak {l})$-invariants with the joint kernel of the operators $e_\delta$ and $f_\delta$. For $q=1$, this definition gives the subspace of $U(\mathfrak {k})$-invariants.

### Proposition 5.1

$\mathcal {A}^\mathfrak {k}_q$ is an associative $U_q(\mathfrak {g})$-algebra with respect to $\star$.

### Proof

Identify $$M_\lambda ^*$$ with $$N_\lambda ^{**}$$ and the locally finite part of $$M_\lambda \otimes M_\lambda ^*$$ with the locally finite part $$\mathrm {End}^\circ _\mathbb {C}(M_\lambda )$$ of $$\mathrm {End}_\mathbb {C}(M_\lambda )$$ regarded as a $$U_q(\mathfrak {g})$$-module. For every completely reducible module *V*, there is a unique $$\tilde{\phi }\in \mathrm {Hom}(V^*,\mathrm {End}_\mathbb {C}^\circ (V))$$ for each $$\phi \in \mathrm {Hom}(M_\lambda \otimes N_\lambda ,V)$$, due to the natural isomorphism of the hom-sets.

Let $\hat{M}_\lambda$ and $\hat{N}_\lambda$ denote the Verma modules, i.e., induced from the $U_q(\mathfrak {b}_\pm )$-modules $\mathbb {C}_{\mp \lambda }$. Every homomorphism $M_{\lambda }\otimes N_{\lambda } \rightarrow V$ amounts to a homomorphism $\hat{M}_{\lambda }\otimes \hat{N}_{\lambda } \rightarrow V$ vanishing on $\sum _{\alpha \in \Pi _\mathfrak {k}}\hat{M}_{\lambda -\alpha }\otimes \hat{N}_{\lambda }+\sum _{\alpha \in \Pi _\mathfrak {k}}\hat{M}_{\lambda }\otimes \hat{N}_{\lambda -\alpha }$. Therefore, $\phi$ corresponds to a unique zero weight element $\Phi (\phi )\in \cap _{\alpha \in \pm \Pi _\mathfrak {k}}\ker e_\alpha = V^\mathfrak {k}$. Given also $\psi \in \mathrm {Hom}(M_\lambda \otimes N_\lambda , W)$ there is a unique element $\phi \circledast \psi \in \mathrm {Hom}(V\otimes W,\mathrm {End}_\mathbb {C}^\circ (M))$ such that $\widetilde{\phi \circledast \psi }=\tilde{\phi }\circ \tilde{\psi }$, where $\circ$ is the multiplication in $\mathrm {End}^\circ (M_\lambda )$. Define $\Phi (\phi )\circledast \Phi (\psi )=\Phi (\phi \circledast \psi )\in (V\otimes W)^\mathfrak {k}$.

Now take $V=W=\mathcal {A}_q$ and $f,g\in \mathcal {A}_q^\mathfrak {k}$ (with respect to the $\triangleright$-action). Then $f\star g$ is the image of $f\circledast g \in (\mathcal {A}_q\otimes \mathcal {A}_q)^\mathfrak {k}$ under the multiplication $\bullet :\mathcal {A}_q\otimes \mathcal {A}_q\rightarrow \mathcal {A}_q$, which is again in $\mathcal {A}_q^\mathfrak {k}$ since $\bullet$ is $\triangleright$-equivariant. Associativity of $\star$ follows from associativity of $\circ$ and $\bullet$. $\square$

### Theorem 5.2

The right $U(\mathfrak {g})$-module $\mathcal {A}^\mathfrak {k}_q$ is a deformation of the $U(\mathfrak {g})$-module $\mathbb {C}[G]^\mathfrak {k}$. The multiplication $\star$ makes $\mathcal {A}^\mathfrak {k}_q$ an associative $U_q(\mathfrak {g})$-algebra, a quantization of $\mathbb {C}[\mathbb {S}^{2n}]$.

### Proof

We only need to make sure that $\mathcal {A}^\mathfrak {k}_q\simeq \oplus _V\> {}^* V^\mathfrak {k}\otimes \!V$ is a deformation of $\mathbb {C}[\mathbb {S}^{2n}]\simeq \mathbb {C}[G]^\mathfrak {k}$. It is done in Proposition 6.2 below. $\square$

Note that, though $\mathcal {A}_q^\mathfrak {k}$ goes over to $\mathbb {C}[G]^\mathfrak {k}$ at $q=1$, the fact $\mathcal {A}_q^\mathfrak {k}\simeq \mathbb {C}[G]^\mathfrak {k}\otimes \mathbb {C}(q)$ as a $\mathbb {C}(q)$-vector space needs a proof because $\ker e_\delta$ and $\ker f_\delta$ may decrease under deformation. That is done in the next section.

## Quantum Euclidian plane

To complete the proof of Theorem 5.2, it is sufficient to check $\dim V_q^\mathfrak {k}=\dim V^\mathfrak {k}$ for all finite-dimensional modules *V* that appear in $\mathbb {C}[\mathbb {S}^{2n}]$. They all can be realized in the polynomial ring of the Euclidian plane $\mathbb {C}^{2n+1}$ [20]. So we are going to look at its quantum version.

Choose a basis $\{x_i\}_{i=-n}^n\subset \mathbb {C}^{N}, N=2n+1$, and define a representation of $U_q(\mathfrak {g})$ on $\mathbb {C}^{N}$ by the assignment

$$\begin{aligned} e_{\alpha _i}\triangleright x_k= \delta _{k,i-1}x_i- \delta _{k,-i}x_{-i+1}, \quad f_{\alpha _i}\triangleright x_k= \delta _{k,i}x_{i-1}- \delta _{k,-i+1}x_{-i} \end{aligned}$$

for $i=1, \ldots ,n$. Then $x_i$ carry weights $\varepsilon _{i}$ subject to $\varepsilon _{i}=-\,\varepsilon _{-i}$. The quantum Euclidian plane $\mathbb {C}_q[\mathbb {C}^{N}]$ is an associative algebra generated by $\{x_i\}_{i=-n}^n$ with relations

$$\begin{aligned}&x_i x_j=q^{-1}x_jx_i, \quad i>j, \quad i\not = \pm j ,\quad x_{1}x_{-1}-x_{-1}x_{1}=(q-1)x_{0}^2,\\&\quad x_{j}x_{-j}-x_{-j}x_{j}=q x_{j-1}x_{-j+1}-q^{-1}x_{-j+1}x_{j-1}, \quad j>1. \end{aligned}$$

They are equivalent to the relations presented in [11].

The representation on $\mathbb {C}^N$ extends to an action $\triangleright$ on $\mathbb {C}_q[\mathbb {C}^N]$ making it a $U_q(\mathfrak {g})$-module algebra. Let $\theta$ denote the involutive algebra and anti-coalgebra linear automorphism of $U_q(\mathfrak {g})$ determined by the assignment $e_{\alpha }\rightarrow - f_{\alpha }$, $q^{h_\alpha }\rightarrow q^{-h_\alpha }$. Define also an anti-algebra linear involution on $\mathbb {C}_q[\mathbb {C}^N]$ by $\iota (x_i)=(x_{-i})$. They are compatible with the action $\triangleright$ in the sense that $\iota (u\triangleright x)= \theta (u)\triangleright \iota (x)$ for all $u\in U_q(\mathfrak {g}), x\in \mathbb {C}_q[\mathbb {C}^N]$.

### Lemma 6.1

For all $k\in \mathbb {Z}_+$ the monomials $x_0^k$ are killed by $e_{\delta }$ and $f_\delta$.

### Proof

Put $c_k=\frac{q^{-k}-1}{q^{-1}-1}$ for $k\in \mathbb {Z}_+$. Since $f_{\alpha _2} x^k_0=0$, the equality $f_{\delta }\triangleright x_0^k=0$ follows from

$$\begin{aligned} f_{\alpha _1}\triangleright x_0^k= & {} -\,x_{-1} x_0^{k-1}c_k,\\ f_{\alpha _1}^2\triangleright x_0^k= & {} x_{-1}^2 x_0^{k-2}qc_{k-1}c_k,\\ f_{\alpha _2}f_{\alpha _1}^2\triangleright x_0^k= & {} -\,x_{-2} x_{-1}x_0^{k-2}c_{k-1}c_k[2]_q,\\ f_{\alpha _1}f_{\alpha _2}f_{\alpha _1}\triangleright x_0^k= & {} -\,x_{-2}x_{-1} x_0^{k-2}c_{k-1}c_k. \end{aligned}$$

Finally, $$e_\delta \triangleright x_0^k=-\iota (f_{\delta }\triangleright x_0^k)=0$$. $$\square $$

The q-version of the quadratic invariant is $C_q=\frac{1}{1+q}x_0^2+\sum _{i=1}^{n}q^{i-1}x_ix_{-i}\in \mathbb {C}_q[\mathbb {C}^N]$. Let $\mathfrak {P}_q^m\subset \mathbb {C}_q[\mathbb {C}^N]$ denote the vector space of polynomials of degree *m* and $\mathfrak {P}_q^m$ the irreducible submodule of harmonic polynomials of degree *m*. Then

$$\begin{aligned} \mathbb {C}_q[\mathbb {C}^N]=\oplus _{m=0}^\infty \mathfrak {P}_q^m, \quad \mathfrak {P}_q^m=\oplus _{l=0}^{[\frac{m}{2}]} C^l_q\mathfrak {H}_q^{m-2l}. \end{aligned}$$

Let $\mathfrak {P}^m$ and $\mathfrak {H}^m$ denote their classical counterparts.

### Proposition 6.2

For any finite-dimensional $U_q(\mathfrak {g})$-module $V_q, \dim V_q^\mathfrak {k}$ is equal to $\dim V^\mathfrak {k}$ of the classical $\mathfrak {k}$-invariants.

### Proof

It is sufficient to show that $\dim (\mathfrak {P}^m_q)^\mathfrak {k}=\dim (\mathfrak {P}^m)^\mathfrak {k}$. In the classical limit, the trivial $\mathfrak {k}$-submodule in $\mathfrak {H}^{m-2l}$ is multiplicity-free, so its dimension in $\mathfrak {P}^m$ is $[\frac{m}{2}]+1$. On the other hand, the subspace of $U_q(\mathfrak {l})$-invariants is spanned by $\{C^l_qx_0^{m-2l}\}_{l=0}^{[\frac{m}{2}]}$ and has the same dimension. Since all $U_q(\mathfrak {l})$-invariants are killed by $e_{\delta }, f_\delta$ by Lemma 6.1, this proves the statement. $\square$

## A

For reader’s convenience, we prove an algebraic identity that is useful for the study of the subalgebras $U_q(\mathfrak {g}_\pm )\subset U_q(\mathfrak {g})$.

### Lemma A.1

Suppose *x*, *y*, *z* satisfy the relations

$$\begin{aligned} \left[ y,[y,x]_q\right] _{\bar{q}}=0,\quad \left[ y,[y,z]_q\right] _{\bar{q}}=0,\quad [x,z]=0. \end{aligned}$$

Then $$\left[ [x,y]_{\bar{q}},[y,z]_q\right] =0$$ and $$[y,[x,[y,z]_q]_{q}]=0.$$

### Proof

The proof is based on the “Jacobi identity”

$$\begin{aligned} \left[ X,[Y,Z]_a\right] _b=\left[ [X,Y]_c,Z\right] _{\frac{ab}{c}}+c\left[ Y,[X,Z]_{\frac{b}{c}}\right] _{\frac{a}{c}}, \end{aligned}$$

which holds true for all elements *X*, *Y*, *Z* of an associative algebra and scalars *a*, *b*, *c* with invertible *c*. Apply it to the equalities

$$\begin{aligned} 0=\left[ x,\left[ y,[y,z]_q\right] _{\bar{q}}\right] _{\bar{q}^2}=\left[ z,\left[ y,[y,x]_q\right] _{\bar{q}}\right] _{\bar{q}^2}=0 \end{aligned}$$

with $a=\bar{q}, b=\bar{q}^2$, $c=\bar{q}$, and rewrite them as

$$\begin{aligned} 0= & {} \left[ [x,y]_{\bar{q}},[y,z]_q\right] _{\bar{q}^2}+\bar{q}\left[ y,\left[ x,[y,z]_{q}\right] _{\bar{q}}\right] _{} = \left[ [z,y]_{\bar{q}},[y,x]_q\right] _{\bar{q}^2}\\&+\,\bar{q}\left[ y,\left[ z,[y,x]_q\right] _{\bar{q}}\right] _{}=0. \end{aligned}$$

Observe that the second terms cancel due to $[x,[y,z]_{q}]_{\bar{q}}=[[x,y]_{\bar{q}},z]_{q}=[z,[y,x]_q]_{\bar{q}}$. Then

$$\begin{aligned} \left[ [x,y]_{\bar{q}},[y,z]_q\right] _{\bar{q}^2} = \left[ [z,y]_{\bar{q}},[y,x]_q\right] _{\bar{q}^2}=\left[ [y,z]_{q},[x,y]_{\bar{q}}\right] _{\bar{q}^2}. \end{aligned}$$

This yields $(1+q^{-2})[[y,z]_{q},[x,y]_{\bar{q}}]=0$, which proves the first formula. Using the “Jacobi identity” with $X=x, Y=y$, $Z=[y,z]_q$, $a=\bar{q}, b=1$, and $c=\bar{q}$, we get

$$\begin{aligned} 0=q\left[ x,[y,[y,z]_q]_{\bar{q}}\right] _{}=q\left[ [x,y]_{\bar{q}},[y,z]_q\right] +\left[ y,[x,[y,z]_{q}]_{q}\right] _{}=\left[ y,\left[ x,[y,z]_{q}\right] _{q}\right] _{}, \end{aligned}$$

which proves the second formula. $\square$
